# Two years COVID-19 pandemic: Development of university students' mental health 2020–2022

**DOI:** 10.3389/fpsyt.2023.1122256

**Published:** 2023-04-06

**Authors:** Elisabeth Kohls, Lukas Guenthner, Sabrina Baldofski, Tanja Brock, Jan Schuhr, Christine Rummel-Kluge

**Affiliations:** ^1^Department of Psychiatry and Psychotherapy, Medical Faculty, Leipzig University, Leipzig, Germany; ^2^Department of Psychiatry and Psychotherapy, University Leipzig Medical Center, Leipzig University, Leipzig, Germany; ^3^Centre for Research, Further Education and Consulting, University of Applied Sciences for Social Work, Education and Nursing Dresden, Dresden, Germany

**Keywords:** COVID-19, university students, mental health, depressive symptoms, alcohol consumption, symptoms of anxiety

## Abstract

**Background:**

The literature indicates a negative impact on the mental health of university students during the COVID-19 pandemic. It remains unclear if this negative impact persists even after lockdown measures are lifted. The current study therefore investigates the mental health status of students by drawing on two previous studies the present study seeks to investigate differences in the mental health status across three time points.

**Methods:**

A cross-sectional, anonymous online survey among students of six universities was conducted between April and May 2022 (*N* = 5,510). Symptoms of depression, anxiety, hazardous alcohol use and eating disorders as well as social and emotional variables were assessed utilizing standardized instruments. Risk- and protective factors for severity of depressive and anxiety symptoms were investigated using multiple regression models. Differences in e.g., symptoms of depression across three time points were assessed with one-way analysis of variance.

**Results:**

More than one third of students exhibited clinically relevant symptoms of depression (35.5%), hazardous alcohol use (33.0–35.5% depending on gender) or anxiety disorder (31.1%). Taken together, almost two out of three (61.4%) students reported clinically relevant symptoms in at least one of the aforementioned symptom patterns, while almost one fifth of students reported suicidal ideation or thoughts of self-harm (19.6%). Higher perceived stress and loneliness significantly predicted higher levels of depressive symptoms, while resilience and social support were identified as protective factors. Compared to 2020 and 2021, levels of depressive symptoms were significantly reduced in 2022, levels of hazardous alcohol consumption showed a small but significant increase from 2021 to 2022. Worryingly, prevalence of suicidal ideation was the highest yet, being significantly higher than in 2020 (14.5%) and 2021 (16.5%).

**Conclusion:**

These results confirm previous results that the pandemic had and still has a negative impact on the mental health of university students. The present study broadens this view by the fact that some areas seem to recover quicker, while others seem to increase worryingly. Especially the persistent rise in suicidal ideation from 2020 to 2021 and to 2022, a constant reduction in reported social support and associated perceived loneliness is concerning. The claim for low-threshold and accessible mental health support for university students remains the same as in the beginning of the pandemic.

## 1. Introduction

Even before the onset of the COVID-19 pandemic, young adults and especially university students were considered to be a vulnerable group for mental disorders, with reports of one in five students suffering from mental health issues (1, 2). Similarly, hazardous alcohol use is known to be prevalent among students in Germany and associated with various negative somatic and mental health outcomes (3, 4). As the peak of onset of most mental disorders is located in teenage years and young adulthood, stressors associated with university life may additionally increase the vulnerability of university students to develop mental health issues. Further, these stressors may lead to a worsening of any existing symptoms with an onset before matriculation, as treatment rates for mental health issues were reported to be low among university students even before the pandemic hit (2, 5).

Beginning at the end of the year 2019, the COVID-19 pandemic made its way across the globe (6). Almost everywhere, people were impacted by the pandemic and the associated government mandated non-pharmaceutical interventions (NPIs), to stop the spreading of the virus. Universities across the globe also had to accommodate these NPIs. As such, much of the academic, occupational, social, and cultural life of students was disrupted. NPIs like government-mandated social distancing and lockdown measures undeniably saved lives and mitigated transmission rates of COVID-19 in the populace (7, 8). However, a number of studies investigating the impact on university students support the assumption that the disruptions of the day-to-day lives caused by the COVID-19 pandemic and the NPIs to control the pandemic may have had a negative impact on various aspects of their mental health (9–13).

Thus, a recent study conducted about a month after England's first national lockdown ended in 2020 found that more than half of students at a university in Northern England reported clinically relevant levels of symptoms of depression and anxiety (12). Another study, conducted by our research group, compared the outcomes of two surveys conducted at Leipzig University, Germany, in the summer of 2020, when the strict social distancing and quarantine measures were eased up (14, 15) and in the spring of 2021, when a federally mandated lockdown was in effect in Germany. The authors found an increase from 2020 to 2021 in the severity of depressive symptoms, suicidal ideation, as well as in alcohol and drug consumption among students (9, 16). Another survey, conducted in the United States, comparing frequencies of mental disorders before and during the pandemic, found evidence for higher frequencies of depression, alcohol use disorder, and bulimia as well as binge eating disorder (10). A systematic review encompassing 16 studies investigating the impact of the pandemic on the mental health of university students identified a relationship between university students' mental health and the onset of the pandemic (11). According to the authors, all studies incorporated in their review indicated that university students reported more feelings of anxiety, depression, fatigue, and distress than before the pandemic. Furthermore, meta-analyses investigating the prevalence of depression and anxiety among university students during the COVID-19 pandemic suggests that up to one third of university students were suffering from symptoms of depression and/or anxiety at some point during the pandemic (13, 17).

Clearly, the majority of the evidence suggests that the pandemic and (while the protective effects of NPIs need to be acknowledged) the associated NPIs designed to curb the spread of COVID-19 have had a negative effect on university students' mental health worldwide. In addition to the severe impact of mental health issues on one's current life, symptoms often recur in the biography of persons who experienced some sort of mental strain earlier in life (18). This adds up to several factors making the treatment of mental health issues a major cost and resource factor within the healthcare system: In Germany, the costs of treatment for depression in 2020 amounted to 9,453 mil. €, while mental disorders caused an outage of work-production depleting 0.4% of the gross domestic income (19, 20).

With the pandemic still ongoing, as of the beginning of April 2022, federally mandated NPIs were lifted in Germany. While there were, and as of the time of writing this article, still are, some NPIs to protect vulnerable groups that may also affect university students (e.g., mask mandates in university buildings and public transportation), there are no strict lockdown measures in effect and much of the academic, occupational, social, and cultural life has since resumed. In general, still little is known if the negative impact of the pandemic on the mental health of university students persists. However, some preliminary evidence suggests that with the ongoing of the pandemic and after strict lockdown measures were lifted, mental health of most students improved (21, 22). To explain the effects of the pandemic and NPIs regarding mental health, the literature suggests resilience, self-efficacy and social-support (23–28) as protective factors. Furthermore, stress and loneliness were indicated to act as risk factors for developing or intensifying symptoms of mental disorders (9, 29–32). Worryingly, recent research conducted by our research group with students from Leipzig University indicated a significant decrease in resilience, social-support, and self-efficacy as well as an increase in perceived stress and loneliness, although effect sizes were small (9, 16).

Additionally, some recent studies suggest a complex relationship between vaccination attitudes and fear of COVID-19 as those who are not willing to get vaccinated reported being less fearful of COVID-19 while receiving a vaccination also seems to reduce fear of COVID-19 (33–36). At the same time, a number of studies suggest that fear of COVID-19 shows associations with outcomes relevant to mental health such as depressive symptoms, anxiety or stress (37, 38).

In the light of past reports of an increase in the occurrence and severity of depressive symptoms, symptoms of generalized anxiety disorder and mixed results concerning hazardous alcohol use as well as general eating disorder psychopathology, the present study aims to (a) gauge the prevalence of those variables in a current post-lockdown convenience sample of German university students.

Further, drawing on the data and results from two congruent cross-sectional surveys (9, 16) conducted in 2020 and 2021, the current paper (b) examines the development of symptoms of depression, suicidal ideation and hazardous alcohol use across three points in time (i.e., 2020, 2021, and 2022). We hypothesized that the elevated levels of depressive symptoms, suicidal ideation and hazardous alcohol and substance use previously identified by Dogan-Sander et al. (9) would significantly decline to levels comparable to those found in the 2020 survey conducted by Kohls et al. (16). Drawing on the additional data sets collected the 2020 and 2021 surveys, (c) differences between the three samples in social and emotional outcomes that are known to act as protective and risk factors were investigated. Specifically it was hypothesized that reduced levels of resilience, social-support and self-efficacy as well as the elevated levels of loneliness and stress would recover to levels similar those found by Kohls (16). Finally, utilizing the current 2022 sample, the present study aims to (d) identify risk- and protective factors for symptoms of depression, symptoms of anxiety, hazardous alcohol use and eating disorder symptomatology.

## 2. Materials and methods

### 2.1. Participants and procedure

The current sample was recruited between April 11th, and May 27th, 2022, at six universities in Saxony, Germany. Participants were recruited using the official university email and social media channels of the universities: students received an email from the administrative office containing a link to the online survey, hosted *via* unipark, an online research platform (www.unipark.de). Two email reminders were sent out approximately 1 week and 4 weeks, respectively, after the initial email invitation. Additionally, an informational video on how to participate in the survey was provided to participating universities. The video was published by three of the participating universities in their social media channels, while the remaining three participating universities did not have their own social media profiles and thus, did not publish the video online. The video itself did not contain a participation link. Rather, the video called attention to the recruitment e-mail, which contained detailed information as well as a link to the survey and was sent to (and only accessible *via*) the students' institutional email addresses.

Criteria for inclusion in the study were current enrollment at one of the universities and being 18 years or older. There were no exclusion criteria. The ethics committee of the Medical Faculty of Leipzig University granted approval for the current study on November 11th, 2021 (file reference: 509/21-ek). All materials used in the study, as well as the declaration of informed consent, were checked and approved by the data protection officer of the Medical Faculty of Leipzig University. Informed consent was provided by all participants before participation in the study *via* an opt-in function at the beginning of the anonymous online survey.

Overall, a total of *N* = 5,510 students completed the online survey. Of these, *n* = 28 (0.5%) participants were excluded in the final analysis because they indicated in a control question that they had not completed the survey conscientiously. Furthermore, *n* = 3 (0.1%) participants were excluded because they were underage and thus, not meeting inclusion criteria, while another *n* = 7 (0.1%) participants were excluded after manually checking the data for implausible cases (*n* = 4 [0.1%] participants indicated studying their current study program for more than 24 semesters and *n* = 3 [0.1%] indicated being aged 80 years or older). All in all, *n* = 5,474 participants were included in the final analysis.

Beside the current sample, the present study takes advantage of two further cross-sectional samples collected by our research group, each including data on surveys with content similar to the present survey: First, during July and August 2020, a survey with *N* = 3,382 students enrolled at Leipzig University, Germany, was conducted to investigate the impact of the pandemic on the mental health status of students at Leipzig University (16). Second, between March and April 2021, another survey with a total of *N* = 5,642 participating students enrolled at Leipzig University, Germany, was conducted to investigate differences in the mental health status of students between 2020 and 2021 (9).

### 2.2. Measures

The current study used a modified version of the online survey utilized in the 2020 and 2021 studies (9, 16). In contrast to the 2020 and 2021 version, items pertaining to aspects of the ongoing pandemic were removed, while other instruments regarding aspects of digitalization (not part of the analysis in the current paper) were added. Furthermore, in the previous surveys the Short Evaluation of Eating Disorders (39) was used for assessment of eating disorder symptoms, in the current survey another instrument was used (see below). The survey was offered in Germany and in English, depending on respondents preferred language.

#### 2.2.1. Sociodemographic information

The survey contained items assessing sociodemographic and socioeconomic information (age, gender, family status, migration status, education, student status [currently enrolled, currently not re-registered or on leave of absence], sources of income, and monthly net income, presence of [chronic] somatic conditions), current vaccination status, and general attitude toward vaccinations (on a 5-point rating scale, ranging from 1 = “opposing” to 5 = “approving”). Parental educational attainment was categorized into four categories (low, middle, elevated, and high attainment) according to the highest educational and professional qualifications of parents (40). Parental educational attainment was considered to be high if both parents were holders of a university degree and considered elevated if only one parent was a holder of a university degree. If both parents completed an apprenticeship, skilled worker's qualification, master craftsman's examination, technical school, or technician's qualification, parental educational attainment was considered to be medium. If only one parent or none of the parents completed such a qualification, parental educational attainment was considered to be low. Migration status was assessed by asking the participants to indicate if they had a migratory background. Response options were to answer the question in the negative, to indicate that they themselves are immigrants, or to indicate that their parents are immigrants.

#### 2.2.2. Mental health measures

**Patient-health-questionnaire-9 (PHQ-9)**. The PHQ-9 (41) utilizes a 4-point rating scale, ranging from 0 = “not at all” to 3 = “nearly every day” to assess the occurrence of depressive symptoms over a 2 week period. More severe depressive symptoms are indicated by a higher total sum score (range: 0-27). Scores of 10 or more are indicative of clinically relevant symptoms (41, 42). Furthermore, suicidal ideation was assessed utilizing item 9 of the PHQ-9, which asks if respondents had any “thoughts that [they] would be better off dead, or of hurting [themselves]” during the past 2 weeks. For the English and for the German instrument, Cronbach's α was .85 and .91, respectively.

**Alcohol use disorder identification test (AUDIT-C)**. The AUDIT-C (43, 44) consists of three items, scored on a 5-point rating scale, evaluating the frequency of alcohol use (0 = “never” to 4 = “4 times a week or more”), the typical quantity being consumed (0 = “1 or 2” to 4 = “10 or more”) as well as the frequency of binge drinking (i. e., consuming more than six alcoholic beverages on a single day; 0 = “never” to 4 = “once per week”). To identify alcohol misuse, a total sum score is computed, with a score of 4 or more indicating alcohol misuse in women, and a score of 5 or more indicating alcohol misuse in men (45). For the English and for the German instrument, Cronbach's α was .76 and .76, respectively.

To evaluate the frequency of drug or substance consumption, a single item adapted based on the structure of AUDIT-C items (“How often do you use drugs/substances?”), rated on a 5-point rating scale (0 = “never” to 4 = “4 times a week or more”) was used.

**Eating disorder examination-questionnaire 8 (EDE-Q8)**. Derived from the long form of the EDE-Q, the EDE-Q8 (46) is a self-report measure to measure general eating disorder pathology. Utilizing eight items, the EDE-Q8 explores different facets of eating disorder psychopathology (e. g., preoccupation with food, discomfort seeing one's own body). The items are rated on a 6-point rating scale, but with differing answer cues depending on the content of the specific item. A scale mean score was computed, with higher scores indicating more severe symptomatology (46). For the English and for the German instrument, Cronbach's α was .92 and .92, respectively.

**General anxiety disorder scale (GAD-7)**. The GAD-7 (47) consists of seven items assessing the frequency of occurring symptoms indicative of general anxiety disorder (GAD) during the previous 2 weeks. The items are rated on a 4-point rating scale (0 = “not at all” to 4 = “nearly every day”) and item scores are added up to compute a total score. According to the authors, a total score of 10 or higher identifies a probable case of GAD (47, 48). For the English and for the German instrument, Cronbach's α was .91 and .87, respectively.

In addition, one single choice item directly assessed if participating students had been diagnosed with a mental disorder in the past (answer options: “yes”, “no”). If participants indicated that they had been diagnosed with a disorder, they were asked to indicate which disorder had been diagnosed (answer options: “depression”, “bipolar disorder”, “anxiety disorder”, “obsessive-compulsive disorder”, “personality disorder”, “eating disorder”, “ADHD/ADD”, and “I don't know”) and for how long they were suffering from each diagnosed disorder (in years). A free text field was also provided if the diagnosed disorder was not listed. Finally, participants were asked if they were currently receiving professional treatment for any mental disorders and if yes, they could indicate if they received medication, psychotherapeutic treatment, both, or neither.

#### 2.2.3. Measures of personal, social, and emotional variables

Data on a selection of personal (resilience, self-efficacy), social (social support), and emotional (perceived stress, loneliness) variables of interest were also collected in the online survey.

**Brief Resilience Scale (BRS)**. While there is no scientific consensus on the definition of the construct of resilience (49, 50), according to the authors of the BRS (51), the ability to “bounce back” and recover from stress represents the original definition of resilience most closely. As such, the instrument is used to assess the ability of individuals to recover from stress using six items, rated on a 5-point Likert scale (1 = “strongly disagree” to 5 = “strongly agree”). A scale mean score is computed, with higher mean scores indicating a more pronounced ability to recover from stress quickly (51, 52). For the English and for the German instrument, Cronbach's α was .77 and .82, respectively.

**General self-efficacy scale (GSE)**. The instrument consist of 10 items rated on a 4-point Likert scale (1 = “not at all true” to 4 = “exactly true”) evaluating general self-efficacy expectations (53). The total sum score reaches from 10 to 40, with higher scores indicating a higher subjective conviction that the individual is able to deal with demanding situations on their own accord (53). For the English and for the German instrument, Cronbach's α was .86 and .87, respectively.

**ENRICHD social support inventory (ESSI)**. To measure the perceived emotional social support, the ESSI consists of five items, scored on a 5-point rating scale (1 = “none of the time” to 5 = “all of the time”) (54, 55). The total score has a range from 5 to 25. Greater levels of perceived social support are indicated by a higher total sum score (54, 55). For the English and for the German instrument, Cronbach's α was .93 and .88, respectively.

**Perceived stress scale (PSS-4)**. A four item instrument assessing perceived stress during the previous month on a 5-point rating scale, ranging from 0 = “never” to 4 = “very often” (56). Item scores are summed up into a total sum score, ranging from 0 to 16. Higher levels of perceived stress are indicated by higher sum scores (54). For the English and for the German instrument, Cronbach's α was .80 and .79, respectively.

**UCLA 3-item loneliness scale**. The three items of the UCLA 3 are rated on a 3-point verbal rating scale (1 = “hardly ever” to 3 = “often”) and summed up into a total sum score, ranging from 3 to 9 (57). The higher the total sum score, the lonelier an individual is considered to be. According to the authors, individuals with a total score of 6 or higher may be considered lonely (57). For the English and for the German instrument, Cronbach's α was .86 and .80, respectively.

### 2.3. Statistical analysis

Statistical analyses were performed using IBM SPSS Statistics version 27.0. A two-tailed α = 0.05 significance level was used for statistical testing. First, descriptive statistics for sociodemographic and socioeconomic variables as well as outcomes regarding mental health, personal, social, and emotional variables were computed. For a better overview of the age distribution in the present sample, age groups were calculated (Table 1). Second, differences between the three separate samples of the surveys conducted in 2020, 2021, and 2022 were investigated. Using χ^2^-tests, differences in categorical outcome variables (i. e. occurrence of mental disorders, suicidal ideation) sociodemographic and socioeconomic variables were analyzed. Where needed, effects found using χ^2^-tests were further decomposed by utilizing the z-test provided by SPSS to compare column proportions. Differences in metric variables (depressive symptoms, harmful alcohol consumption, perceived stress, resilience, social support, self-efficacy, loneliness) were assessed using one-way analysis of variance (ANOVA), followed by Tukey *post-hoc* tests to decompose significant effects. While sample sizes differed between the three surveys, the variance ratio for all variables of interest remained below 1.5 in all comparisons between groups. As such we remained confident in relying on ANOVA to compute group comparisons (58). Taking multiple testing into account, Bonferroni correction was used where needed. $\eta {{}^{\text{2}}}_{\text{partial}}$ was interpreted as small when $\eta {{}^{\text{2}}}_{\text{partial}}$ =0.001, as medium when $\eta {{}^{\text{2}}}_{\text{partial}}$ =0.06 and as large when $\eta {{}^{\text{2}}}_{\text{partial}}$ =0.14 (59). A comparison of GAD-7 and EDE-Q8 mean scores of the current and previous surveys was not possible, as those instruments were only utilized in the 2022 survey. As the surveys in 2020 and 2021 were exclusively conducted with students studying at Leipzig University, all comparisons were repeated including only students from Leipzig University to check for differences in the results.

**Table 1:** Characteristics of the sample.

	**(*n*, %)**	**Depressive symptoms**	**Perceived stress**	**Social support**	**Loneliness**	**Self-efficacy**
		**M(SD)**	**M(SD)**	**M(SD)**	**M(SD)**	**M(SD)**
**Total**	5474	8.34 (5.51)	7.23 (3.20)	20.71 (4.10)	5.75 (1.86)	2.77 (0.46)
**Gender**						
Female	3775 (69.0%)	8.64 (5.47)	7.43 (3.13)	21.19 (3.79)	5.78 (1.84)	2.73 (0.45)
Male	1599 (29.2%)	7.35 (5.38)	6.64 (3.26)	19.70 (4.54)	5.63 (1.90)	2.87 (0.47)
Diverse	100 (1.8%)	13.36 (5.62)	9.19 (3.00)	18.68 (19.50)	6.55 (1.94)	2.47 (0.49)
**Age**						
< 20	620 (11.3%)	8.51 (5.47)	7.17 (3.05)	20.72 (3.89)	5.93 (1.74)	2.76 (0.44)
20–25	3620 (66.1%)	8.42 (5.53)	7.27 (7.00)	20.84 (4.03)	5.79 (1.87)	2.75 (0.46)
26–30	772 (14.1%)	8.34 (5.63)	7.31 (3.30)	20.66 (4.12)	5.61 (1.86)	2.82 (0.47)
≥31	462 (8.4%)	7.60 (5.26)	6.91 (3.28)	19.78 (4.68)	5.43 (1.90)	2.84 (0.47)
**Relationship status**						
In a relationship	2668 (48.7%)	7.82 (5.28)	7.07 (3.14)	22.21 (3.06)	5.43 (1.79)	2.79 (0.45)
Single	2806 (51.3%)	8.85 (5.69)	7.39 (3.25)	19.28 (4.44)	6.05 (1.87)	2.75 (0.47)
**Residential status**						
Alone	1355 (24.8%)	8.94 (5.70)	7.46 (3.24)	19.71 (4.59)	6.06 (1.87)	2.78 (0.48)
Shared	4119 (75.2%)	8.15 (5.44)	7.16 (3.18)	21.04 (3.87)	5.65 (1.85)	2.77 (0.46)
**Being parent**						
Yes	316 (5.8%)	7.21 (5.15)	6.72 (3.17)	20.73 (4.35)	5.28 (1.87)	2.90 (0.44)
No	5158 (94.2%)	8.42 (5.53)	7.27 (3.20)	20.71 (4.08)	5.78 (1.86)	2.76 (0.46)
**Migration status**						
Self	338 (6.2%)	10.02 (6.43)	8.25 (3.25)	18.63 (5.09)	6.26 (2.02)	2.79 (0.51)
Parents	362 (6.6%)	9.47 (5.68)	7.68 (3.31)	20.18 (4.23)	5.86 (1.93)	2.73 (0.45)
No migration background	4774 (87.2%)	8.14 (5.40)	7.13 (3.17)	20.90 (3.96)	5.70 (1.84)	2.77 (0.46)
**Parental education**						
Low	191 (3.5%)	10.42 (6.43)	8.03 (3.10)	18.71 (5.18)	6.38 (1.89)	2.72 (0.49)
Middle	1893 (34.6%)	8.32 (5.34)	7.30 (3.22)	20.71 (4.06)	5.70 (1.83)	2.74 (0.45)
Elevated	1357 (24.8%)	8.03 (5.27)	7.05 (3.17)	20.91 (3.87)	5.72 (1.84)	2.79 (0.45)
High	1694 (30.9%)	8.15 (5.55)	7.06 (3.16)	20.96 (4.03)	5.69 (1.89)	2.80 (0.47)
Don't know/Prefer not to say	339 (6.2%)	9.58 (6.31)	8.00 (3.28)	19.78 (4.40)	6.06 (1.90)	2.69 (0.50)
**Current income**						
No income	556 (10.2%)	9.06 (5.96)	7.74 (3.27)	20.00 (4.65)	5.94 (1.87)	2.69 (0.49)
0–499 €/mo	1048 (19.1%)	8.73 (5.68)	7.58 (3.18)	20.77 (4.15)	5.92 (1.87)	2.72 (0.47)
500–999 €/mo	2383 (43.5%)	8.38 (5.40)	7.27 (3.19)	20.71 (4.00)	5.80 (1.84)	2.76 (0.45)
≥1,000 €/mo	1343 (24.5%)	7.71 (5.33)	6.70 (3.12)	20.98 (3.96)	5.44 (1.86)	2.86 (0.45)
Prefer not to say	144 (2.6%)	8.22 (5.72)	7.22 (3.22)	20.46 (4.05)	5.82 (1.83)	2.77 (0.49)
**Study program**						
Bachelor	1928 (35.2%)	9.14 (5.74)	7.62 (3.22)	20.29 (4.25)	6.02 (1.86)	2.69 (0.48)
Master	3108 (56.8%)	7.98 (5.37)	7.06 (3.18)	20.87 (4.04)	5.64 (1.85)	2.81 (0.45)
Other	438 (8.0%)	7.48 (5.15)	6.79 (3.00)	21.41 (3.63)	5.38 (1.78)	2.86 (0.41)
**Diagnosed mental disorder**						
Yes	1239 (22.6%)	11.18 (5.99)	8.42 (3.28)	19.90 (4.33)	6.34 (1.83)	2.57 (0.49)
No	4235 (77.4%)	7.51 (5.08)	6.89 (3.09)	20.94 (4.00)	5.58 (1.84)	2.83 (0.44)
**Somatic condition**						
Yes	933 (17.0%)	9.70 (5.91)	7.78 (3.33)	20.80 (4.20)	6.02 (1.87)	2.71 (0.50)
No	4541 (83.0%)	8.07 (5.39)	7.12 (3.16)	20.81 (4.07)	5.69 (1.85)	2.78 (0.46)
**Vaccination status**						
Fully vaccinated	5102 (93.2%)	8.37 (5.55)	7.23 (3.20)	20.71 (4.09)	5.75 (1.86)	5.75 (1.86)
Intends to vaccinate	59 (1.1%)	8.03 (4.70)	7.37 (3.47)	20.42 (4.44)	6.14 (1.92)	6.14 (1.92)
Not vaccinated and no intention to do so	313 (5.7%)	7.95 (5.11)	7.25 (3.15)	20.75 (4.22)	5.66 (1.87)	5.66 (1.87)

To evaluate predictors of mental health outcomes in the current sample collected in 2022, multiple regression analyses were performed for PHQ-9 scores, AUDIT-C scores, EDE-8 scores and GAD-7 scores as dependent variables. Continuous predictors included in the models were age, resilience, self-efficacy, perceived social support, loneliness and stress, as well as general vaccination attitude. Categorical predictors included in the models were gender, being a parent, residential status, marital status, migrant background, income, parental education, presence of (chronic) somatic conditions and mental disorders, as well as vaccination status. For vaccination status, being vaccinated was chosen as reference category, as it was suspected that most participants were already vaccinated. Categorical predictors were dummy coded as needed. All VIF were < 10, as such confirming the assumption that independent variables did not exhibit multicollinearity in the current analysis.

## 3. Results

### 3.1. Sociodemographic characteristics

Sociodemographic characteristics and descriptive results of the PHQ-9, PSS-4, ESSI, UCLA, and GSE sum scores of the present sample are shown in Table 1. In the present sample, the mean age was 23.71 years (*SD* = 4.81), with participants ranging from 18 to 56 years old. Regarding gender, *n* = 3,775 (69.0%) of the participants indicated being female, *n* = 1,599 (29.2%) being male, and *n* = 100 (1.8%) being diverse. In addition, *n* = 933 (17.0%) of participants indicated suffering from a (chronic) somatic condition. In the present sample, participants were generally in favor of vaccinations, with a mean score of 4.63 (*SD* = 0.78) regarding their general attitude toward vaccinations and a total of *n* = 5,102 (93.2%) of participants reporting that they were fully vaccinated against COVID-19. Furthermore, *n* = 59 (1.1%) participants stated that they were not fully vaccinated but wanted to do so and *n* = 313 (5.7%) indicated that they were not vaccinated and had no intention of doing so. Compared with the 2020 and 2021 samples, while no differences were found in gender χ^2^_(2, 14498)_ = 5.98, *p* = < 0.001, there was a significant difference in age, *F*_(2, 14494)_ = 13.41, *p* < 0.001, between the samples. A Tukey *post-hoc* test indicated, that compared to 2020 (age M = 23.98, SD = 4.64) participants in 2021 (age M = 23.47, SD = 4.46), mean difference (MD) = 0.52, 95%-CI (0.28, 0.76), *p* < 0.001, and 2022, MD = 0.28, 95%-CI (0.04, 0.51), *p* = 0.018, were significantly younger, while participants in 2021 were significantly younger when compared to participants in 2022, MD = −0.24, 95%-CI (−0.45, 0.04), *p* = 0.016.

### 3.2. Mental health outcomes and comparisons to previous survey results from 2020 and 2021

All in all, 22.6% (*n* = 1,239) of participants disclosed that they had previously been diagnosed with a mental disorder and less than half of those (47.7%; *n* = 591) reported receiving any form of treatment (i. e., psychotherapy and/or medication) at the time of the survey. The three most frequent self-reported mental disorder diagnoses among the sample were depression (11.2%; *n* = 612), anxiety disorder (8.0%; *n* = 437), and eating disorders (3.9%; *n* = 215). In the current survey of 2022, nearly two-thirds (61.4%, n = 3363) of participants met the cut-off for clinically significant symptom severity on at least one of the instruments (PHQ-9, AUDIT-C, GAD-7) that specify such cut-offs.

Regarding depressive symptoms, the mean sum score of the PHQ-9 was 8.34 (*SD* = 5.51), with *n* = 1,936 (35.5%) participants exhibiting a score of 10 or higher, indicating clinically relevant symptom severity. When compared to previous survey data from 2020 and 2021, significant differences in the mean PHQ-9 scores were found (Table 2). Results of the *post-hoc* analysis show that participants reported significantly less severe levels of depressive symptoms in 2020 than in 2021, MD = −1.43, 95%-CI (−1.72, −1.14), *p* < 0.001, and significantly more than in 2022, MD = 0.31, 95%-CI (0.03,0.60), *p* < 0.029. In 2021, participants reported significantly more severe levels of depressive symptoms than in 2022, MD = 1.74, 95%-CI (1.49, 1.99), *p* < 0.001. When repeating this comparison analyzing only participants studying at Leipzig University, results differed. While the overall difference remained significant, *F*_(2, 11983)_ = 103.27, *p* < 0.001, $\eta {{}^{\text{2}}}_{\text{partial}}$ = 0.017, a *post-hoc* analysis did not find a significant difference in mean scores between the surveys conducted in 2020 and 2022, MD = 0.12, 95%-CI (−0.12, 0.46), *p* = 0.666, while significant differences emerged when comparing 2020 and 2021, MD = 1.43, 95%-CI (−1.72, 1.14), *p* < 0.001, as well as 2021 and 2022, MD = 1.55, 95%-CI (1.25, 1.85), *p* < 0.001.

**Table 2:** Comparison of mean scores between surveys conducted in 2020, 2021, and 2022.

	**Survey 2020 (*N* = 3,382) *M (SD)***	**Survey 2021 (*N* = 5,642)** ***M (SD)***	**Survey 2022 (*N* = 5,474) *M (SD)***	**Result**	***p***	** $\eta_{\text{partial}}^{2}$ **
PHQ-9	8.66 (5.46)^a^	10.09 (5.84)^b^	8.35 (5.52)^c^	*F*_(2, 14495)_ = 146.69	< 0.001	0.020
AUDIT-C	2.87 (2.16)^a, b^	2.76 (2.19)^b^	2.91 (2.31)^a, c^	*F*_(2, 14495)_ = 6.51	0.002	0.001
BRS	3.16 (0.76)^a^	3.10 (0.76)^b^	3.04 (0.77)^c^	*F*_(2, 14438)_ = 27.47	< 0.001	0.004
ESSI	21.40 (3.70)^a^	20.83 (3.91)^b^	20.71 (4.10)^b^	*F*_(2, 14495)_ = 34.76	< 0.001	0.005
GSE	2.86 (0.45)^a^	2.80 (0.45)^b^	2.77 (0.46)^c^	*F*_(2, 14495)_ = 39.70	< 0.001	0.005
PSS-4	7.35 (3.17)^a^	7.88 (3.08)^b^	7.23 (3.20)^a^	*F*_(2, 14438)_ = 64.53	< 0.001	0.009
UCLA	4.54 (1.59)^a^	6.37 (1.8)^b^	5.75 (1.86)^c^	*F*_(2, 14495)_ = 1114.04	< 0.001	0.133

PHQ-9, Patient-Health-Questionnaire 9; AUDIT-C, Alcohol Use Disorders Identification Test; BRS, Brief Resilience Scale; ESSI, ENRICHD Social Support Inventory; GSE, General Self-Efficacy Scale; PSS-4, Perceived Stress Scale; UCLA, Three-Item Loneliness Scale; cells with the same superscript in a row did not differ significantly.

Significant differences in the occurrence of mental disorders, $\chi_{\left(2,14444\right)}^{2}$ = 21.07, *p* < 0.001 were found between the three surveys. The proportion of participants stating that they had been diagnosed with a mental disorder in the past was significantly higher, *p* < 0.05, in 2022 (*n* = 1239, 41.5%) than in 2021 (*n* = 1105, 37.0%) and in 2020 (*n* = 644, 21.6%). Furthermore, *n* = 1,075 (19.6%) of the current sample indicated suicidal ideation or thoughts of self-harm in the 2 weeks preceding the survey, a significantly higher proportion than in both other samples (2020: *n* = 490, 14.5%; 2021: *n* = 936, 16.6%), $\chi_{\left(2,14498\right)}^{2}$ = 38.11, *p* < 0.001. Repeating these analyses only using participants from Leipzig University, results remained the same.

The GAD-7 mean sum score was 7.76 (SD = 4.79), with *n* = 1,704 (31.1%) participants reaching the cut-off for clinically relevant symptom severity. Additionally, the mean sum score of the EDE-Q8 was 1.62 (SD = 1.49) (46).

Concerning alcohol consumption, *n* = 1,244 (33.0%) of female participants and *n* = 568 (35.5%) of male participants reached scores above the cut-off for alcohol misuse. In the diverse subgroup, *n* = 39 (39.0%) scored above the cut-off for women. This proportion declined to *n* = 32 (32.0%) when using the cut-off for males. While statistical significant differences in AUDIT-C scores between the surveys were found (Table 2), *post-hoc* analyses only identified a significant difference between the years 2021 and 2022, MD = −0.15, 95%-CI (−0.25, −0.05), *p* = 0.001, such that participants in 2021 reported significantly less alcohol use than in 2022. When repeating this comparison analyzing only participants studying at Leipzig University, results remained the same.

Finally, with reference to drug and substance consumption, *n* = 4,720 (86.2%) of participants in the 2022 survey reported having never consumed any drugs or substances, with a score of *M* = 1.24 (*SD* = 0.71) regarding the frequency of drug consumption. In comparison to the data of the previous surveys, significant differences in the frequency of drug consumption were found, *F*_(2, 14440)_ = 22.73, *p* < 0.001, $\eta {{}^{\text{2}}}_{\text{partial}}$ = 0.003, with *post-hoc* analysis indicating that in the 2022 survey, participants reported significantly less frequent drug consumption than participants in the 2020, MD = −0.06, 95%-CI (−0.10, −0.02), *p* = 0.002, and 2021, MD = −0.10, 95%-CI (−0.14, −0.06), *p* < 0.001, surveys. When repeating this comparison analyzing only participants studying at Leipzig University, the effect remained significant, F_(2, 11928)_ = 10.73, *p* < 0.001, η^2^_partial_ =0.002, but there was no significant difference between 2022 and 2020, MD = −0.04, 95%-CI (−0.09,0.01), *p* = 0.170, yet the difference between 2022 and 2021 remained significant, MD = −0.08, 95%-CI (−0.13, −0.04), *p* < 0.001.

### 3.3. Social and emotional outcomes and comparisons to previous survey results from 2020 and 2021

The results of the comparison of social and emotional outcomes between the three surveys are displayed in Table 2. Results imply that the scores of resilience (BRS), social support (ESSI), general self-efficacy (GSE), perceived stress (PSS-4) as well as loneliness (UCLA) differed significantly between the three surveys. Participants in 2020 indicated to be significantly more resilient than in 2021 MD = 0.06, 95%-CI (0.02, 0.10), *p* < 0.001, or 2022, MD = 0.12, 95%-CI (0.08, 0.16), *p* < 0.001, while participants in 2021 reported being significantly more resilient than in 2022, MD = 0.06, 95%-CI (0.03, 0.10), *p* < 0.001. Repeating these analyses only using participants from Leipzig University, results did not differ.

Regarding self-efficacy, participants in 2020 reported significantly more self-efficacy than in 2021 MD = 0.05, 95%-CI (0.03, 0.08), *p* < 0.001, or 2022, MD = 0.08, 95%-CI (0.06, 0.11), *p* < 0.001, while participants in 2021 reported significantly more self-efficacy than in 2022, MD = 0.03, 95%-CI (0.01, 0.05), *p* < 0.001. A repetition of the analyses only utilizing participants from Leipzig University did not lead to different results.

Investigating social support (ESSI), Tukey's *post-hoc* test indicated significant differences (both *p* < 0.001) between the ESSI scores of the years 2020 and 2021, MD = 0.57, 95%-CI (0.37, 0.77), as well as 2020 and 2022 (MD = 0.69, 95%-CI (0.49, 0.90), while the difference between 2021 and 2022, MD = 0.12, 95%-CI (−0.05, 0.30) did not reach statistical significance (*p* = 0.123). As before, results remained the same when repeating the analysis only utilizing participants from Leipzig University.

In terms of perceived stress, participants in 2020 indicated to be significantly less stressed than in 2021 MD = −0.53, 95%-CI (−0.70, −0.37), *p* < 0.001, while no significant difference emerged when compared to 2022, MD = 0.11, 95%-CI (−0.05, 0.28), *p* = 0.286. Participants in 2021 reported being significantly more stressed than in 2022, MD = 0.65, 95%-CI (0.50, 0.79), *p* < 0.001. Again, the pattern of effects remained the same, when repeating the analysis only utilizing participants from Leipzig University.

On the subject of perceived loneliness, participants in 2020 reported to be significantly less lonely than in 2021 MD = −1.82, 95%-CI (−1.92, −1.73), *p* < 0.001, or 2022, MD = −0.12, 95%-CI (−1.30, 1.11), *p* < 0.001, while participants in 2021 reported being significantly more lonely than in 2022, MD = 0.62, 95%-CI (0.54, 0.70), *p* < 0.001. A repetition of the analysis only utilizing participants from Leipzig University showed the same pattern of significant differences.

### 3.4. Predictors of mental health outcomes

Results of the regression analysis investigating predictors of depressive symptoms are displayed in Table 3. Results indicate that the regression model explained 56% of the variance, *R*^2^ = 0.56, *F*_(25, 5399)_ = 277.63, *p* < 0.001. Being gender diverse (*p* < 0.001), having parents with a migrant background (*p* = 0.012) suffering from (chronic) somatic disorders (*p* < 0.001), suffering from mental disorders (*p* < 0.001) and higher levels of perceived loneliness and stress (both *p* < 0.001) were predictors of higher levels of depressive symptoms. Being older age, (*p* = 0.001), having parents with an elevated educational background (*p* = 0.036) and higher levels of resilience, self-efficacy and social support (all *p* < 0.001) were identified as protective factors.

**Table 3:** Linear regression analysis for predictors of depressive symptomatology measured by PHQ-9 (Patient Health Questionnaire-9).

	**Unstandar-dized B**	**Standardized Beta**	***T***	***p***	**95% CI**	
					**lower**	**upper**
(constant)	10.88		9.35	0.000	8.60	13.16
Age	−0.04	−0.04	−3.41	0.001	−0.07	−0.02
**Gender (ref: female)**						
Male	−0.23	−0.02	−1.94	0.052	−0.45	0.00
Diverse	1.69	0.04	4.52	0.000	0.96	2.43
Being a parent (ref: yes)	−0.15	−0.01	−0.56	0.577	−0.66	0.37
Residential status (ref: alone)	−0.11	−0.01	−0.95	0.341	−0.35	0.12
Marital status (ref: in a relationship)	0.12	0.01	1.11	0.267	−0.10	0.34
**Migrant background (ref.: no migrant background)**						
Self	0.41	0.02	1.88	0.060	−0.02	0.83
Parents	0.51	0.02	2.52	0.012	0.11	0.91
**Income (ref.: no own income)**						
0–499€	−0.09	−0.01	−0.47	0.638	−0.47	0.29
500–999€	−0.07	−0.01	−0.43	0.669	−0.42	0.27
>1,000€	0.27	0.02	1.41	0.158	−0.11	0.65
Prefer not to say	−0.11	0.00	−0.32	0.751	−0.79	0.57
**Parental education (ref.: low)**						
Middle	−0.35	−0.03	−1.88	0.060	−0.72	0.01
Elevated	−0.40	−0.03	−2.10	0.036	−0.78	−0.03
High	−0.25	−0.02	−1.35	0.178	−0.62	0.12
Chronic somatic conditions (ref.: yes)	−0.56	−0.04	−4.19	0.000	−0.83	−0.30
Mental disorders (ref.: yes)	−1.19	−0.09	−9.34	0.000	−1.45	−0.94
BRS	−0.83	−0.12	−9.61	0.000	−1.00	−0.66
GSE	−0.57	−0.05	−3.87	0.000	−0.86	−0.28
ESSI	−0.10	−0.07	−6.38	0.000	−0.13	−0.07
UCLA	0.44	0.15	13.16	0.000	0.37	0.50
PSS−4	0.84	0.48	39.64	0.000	0.79	0.88
**Vaccination status (ref.: yes)**						
Intention to	−0.77	−0.01	−1.55	0.122	−1.74	0.20
No	−0.27	−0.01	−1.09	0.276	−0.76	0.22
General vaccination attitude	−0.04	0.00	−0.48	0.629	−0.18	0.11

Regression sample size *n* = 5,474. PHQ-9, Patient-Health-Questionnaire 9; AUDIT-C, Alcohol Use Disorders Identification Test; BRS, Brief Resilience Scale; ESSI, ENRICHD Social Support Inventory; GSE, General Self-Efficacy Scale; PSS-4, Perceived Stress Scale; UCLA, Three-Item Loneliness Scale.

Concerning general anxiety disorder symptomatology, results are displayed in Table 4. 53% of the variance in the outcome variable, *R*^2^ = 0.53, *F*_(25, 5399)_ = 245.02, *p* < 0.001, could be explained by the regression model. Higher levels of general anxiety disorder symptomatology were predicted by having an income of >1,000€ (when compared to not having an own income; *p* = 0.010), suffering from a (chronic) somatic condition (*p* < 0.001), suffering from a mental disorder (*p* < 0.001) and higher levels of loneliness and perceived stress (both *p* < 0.001). Being of older age (*p* = 0.012), being male (when compared to being female; *p* = 0.003), being single (*p* = 0.002), higher levels of resilience (*p* < 0.001) as well as reporting to not be vaccinated predicted lower levels of general anxiety disorder symptomatology.

**Table 4:** Linear regression analysis for predictors of generalized anxiety disorder symptoms by GAD-7 (General Anxiety Disorder scale 7).

	**Unstandar-dized B**	**Standardized Beta**	***T***	***p***	**95% CI**	
					**Lower**	**Upper**
(constant)	8.47		8.10	0.000	6.42	10.52
Age	−0.03	−0.03	−2.52	0.012	−0.05	−0.01
**Gender (ref: female)**						
Male	−0.32	−0.03	−3.02	0.003	−0.52	−0.11
Diverse	0.42	0.01	1.24	0.214	−0.24	1.08
Being a parent (ref: yes)	−0.05	0.00	−0.20	0.841	−0.51	0.41
Residential status (ref: alone)	0.06	0.01	0.56	0.578	−0.15	0.27
Marital status (ref: in a relationship)	−0.31	−0.03	−3.10	0.002	−0.51	−0.11
**Migrant background (ref.: no migrant background)**						
Self	0.33	0.02	1.72	0.086	−0.05	0.71
Parents	0.07	0.00	0.36	0.720	−0.29	0.42
**Income (ref.: no own income)**						
0–499€	−0.12	−0.01	−0.67	0.505	−0.46	0.22
500–999€	0.00	0.00	−0.02	0.987	−0.31	0.30
>1,000€	0.44	0.04	2.57	0.010	0.10	0.78
Prefer not to say	0.28	0.01	0.88	0.377	−0.34	0.89
**Parental education (ref.: low)**						
Middle	0.00	0.00	0.00	0.997	−0.33	0.33
Elevated	−0.11	−0.01	−0.61	0.542	−0.45	0.23
High	0.02	0.00	0.11	0.910	−0.31	0.35
Chronic somatic conditions (ref.: yes)	−0.28	−0.02	−2.31	0.021	−0.52	−0.04
Mental disorders (ref.: yes)	−0.95	−0.08	−8.24	0.000	−1.17	−0.72
BRS	−1.33	−0.22	−17.20	0.000	−1.49	−1.18
GSE	−0.19	−0.02	−1.40	0.161	−0.45	0.07
ESSI	0.01	0.01	0.65	0.518	−0.02	0.04
UCLA	0.34	0.13	11.26	0.000	0.28	0.39
PSS−4	0.71	0.47	37.26	0.000	0.67	0.74
**Vaccination status (ref.: yes)**						
Intention to	−0.43	−0.01	−0.96	0.338	−1.30	0.45
No	−0.44	−0.02	−1.97	0.049	−0.87	0.00
General vaccination attitude	0.00	0.00	−0.02	0.983	−0.13	0.13

Regression sample size *n* = 5,474. PHQ-9, Patient-Health-Questionnaire 9; AUDIT-C, Alcohol Use Disorders Identification Test; BRS, Brief Resilience Scale; ESSI, ENRICHD Social Support Inventory; GSE, General Self-Efficacy Scale; PSS-4, Perceived Stress Scale; UCLA, Three-Item Loneliness Scale.

Finally, regarding harmful alcohol use, only 7% of the variance, *R*^2^ = 0.07, *F*_(25, 5399)_ = 16.50, *p* < 0.001, in the outcome variable could be explained by the regression model. Furthermore, as only 15% of the variance, *R*^2^ = 0.15, *F*_(25, 5399)_ = 39.45, *p* < 0.001, could be explained by the regression model predicting eating disorder symptomatology, we elected to omit further interpretation of these models.

## 4. Discussion

The present study examined the occurrence and severity of depressive symptoms, symptoms of generalized anxiety disorder, hazardous alcohol use as well as general eating disorder psychopathology among university students. Further, risk and protective factors relevant for students' mental health in a current post-lockdown sample of German university students were explored. Ultimately, the development of symptoms of mental health disorders in university students had been analyzed, drawing on three separate samples collected across three time points (2020, 2021, 2022).

The results from the present study confirm the picture from the international literature that the pandemic has overall a negative impact on the mental health of university students- and that this picture overall seems to persist, with a trend to decrease in some areas. Nevertheless, the hypothesized decline of elevated levels of depressive symptoms and hazardous alcohol use previously identified by Dogan-Sander et al. (9) could be confirmed by the present study.

### 4.1. Mental health outcomes and comparisons to previous survey results from 2020 and 2021

Taken together, almost one in three (61.4%) students reached the cut-off for clinically relevant symptom severity on at least one of the instruments (PHQ-9, AUDIT-C, or GAD-7) that specified such cut-off values. While the EDE-Q8 does not specify cut-off values, the sample mean roughly corresponds to the 78th percentile in a sample of the general population, which may indicate an elevated level of eating disorder symptoms in the present sample (46). It is important to highlight that those instruments are self-report-based screening instruments to determine specific symptom loads (i.e., depressive symptoms, general anxiety symptoms, and alcohol misuse or dependency symptoms) and are not a clinical diagnosis or an indication for further treatment by themselves. Nevertheless, this result is a clear indication of a worrying level of psychological distress and clinically relevant symptom load among students in the present sample. More specifically, a substantial number of students still exhibited clinically relevant symptoms of depression in 2022 (*n* = 1,936; 35.5%). The levels of depressive symptoms were significantly reduced in 2022, when compared to the 2021 sample. Although it has to be kept in mind that the current study draws on three separate cross-sectional samples, it can be summarized, that the overall trend seems to be slightly positive, rather than sustained negative regarding depressive symptoms in university students in Germany over time. Nevertheless, more than one third of students report clinically relevant depressive symptoms, which is still a very worrying result. This result is also in line with several other studies among university students in other countries (60–63).

Further, nearly one fourth (*n* = 1,239; 22.6%) of participants reported that they had been diagnosed with a mental disorder and less than half of those (47.7%; *n* = 591) reported not to receive any form of treatment. This result has remained unchanged over time and may indicate persistent barriers to seeking help or to accessing mental health care in university students (9). However, it is also possible that participants had received a diagnosis in their lifetime but were no longer in need of professional treatment at the time when the survey was conducted. That being said, past studies in the U.S. indicate low treatment rates as well as significant barriers to accessing mental health care among university students (2, 5, 64). As such, further research seems warranted to evaluate treatment needs in German university students.

Although the effect size was small, hazardous alcohol consumption significantly increased, when comparing the 2021 and 2022 samples, while no difference emerged when comparing the 2020 and 2022 samples. Because even minor differences can produce statistically significant results in large samples, caution is advised when interpreting such effects. As such, it is unclear whether the found reported effect can be interpreted as a meaningful change in consumption behavior. So far, very heterogeneous results had been reported in the international literature regarding alcohol consumption among students during the COVID-19 pandemic ranging from a clear increase, decrease or unchanged status (65–70). This picture seems to continue in 2022, supported by the present results and it might need further studies to be able to draw a more comprehensive picture and understand all related factors.

Worryingly, the prevalence of suicidal ideation was the highest in the 2022 survey, with 19.6% of all participants indicating suicidal ideation or thoughts of self-harm in the 2 weeks preceding the survey, which was significantly higher than in the 2020 (14.5%) sample and the 2021 (16.5%) sample. This worrying increase of an already high level of suicidal ideation among students measured by the PHQ-9 cannot be sufficiently explained by the analyzed data of this survey, but the magnitude is comparable with other recent results from other countries (71). It may be explained by the fact that, during the pandemic outbreak, death became a more salient topic in everyday life, communication, and in all media contents. Additionally, a large proportion of the population experienced the loss of family members or beloved ones among the over 150,000 deaths due to COVID-19 in Germany (72). Nevertheless, recent studies indicate that an increased salience of death due to the COVID-19 pandemic would also be associated with an increase in the level of depressive symptoms, which was not the case in the present study (73–75). Additionally, Russia's invasion of Ukraine, which started in February 2022, the recent energy crisis, economic changes, rising inflation rates, climate change and related worry in youths and adolescents (76–78) could, to some extent, contribute to an explanation of this specific increase in only suicidal ideation, but further research is needed.

It might also be important to take aspects of loneliness and social isolation into account to potentially explain the recent results regarding suicidal ideation in university students. Feeling alone and a lack of social contacts can cause several uncomfortable feelings and tension- and could additionally be understood as a lack of distraction from suicidal thoughts (if already present) and yield in higher rumination behavior. Following this, coronavirus-related NPIs could be understood as making vulnerable people even more vulnerable to suicidal thoughts and reduce the desire to live (79). Still, it remains unclear, if suicidal ideation (in the current situation after 2 years of pandemic and several lockdowns) comports with higher depressive symptoms, or not. Some studies report it does (triggered by various risk factors such as stress, fears of contracting the infection from others, financial instability) (80–84), some does, it might occur (and change) independently (85)—as in the present study.

### 4.2. Social and emotional outcomes and comparisons to previous survey results from 2020 and 2021

Results of the present analysis implied that scores for resilience, social support, general self-efficacy, perceived stress, and loneliness differed significantly between the three surveys.

The scores for social support, self-efficacy, and resilience all showed a continuous decrease, when comparing the 2020, 2021, and 2022 in chronological order. The reported levels of social support and loneliness by the university students did not (fully) recover in the 2022 sample, when compared to the 2020 sample, which might be explained by a sustainable loss of relationships due to extended lock-down periods and phases of social isolation, which is also in line with the results of a recent longitudinal study across several countries (86).

There were significant differences between the reported scores for resilience, self-efficacy and perceived stress across the three samples in university students. However, the directions of these changes were not consistent, and interpretability remains limited because the present study is not longitudinal and resilience and self-efficacy are generally considered rather stable traits. There is consensus that resilience and self-efficacy are important protective factors and associated with positive mental health (26–28), but it remains unclear if and where changes might occur due to the pandemic.

### 4.3. Predictors of mental health outcomes

In the present analysis, while controlling for a past diagnosis of mental disorders, higher perceived stress and loneliness significantly predicted higher levels of depressive symptoms, while resilience and social support were identified as protective factors- which is in line with previous results from the other time points (9, 16). However, results concerning the role of age and gender seem inconsistent in the literature: gender was either found to be a significant predictor limited to symptoms of anxiety (in U.S. students) (87) and panic but not for depression (in German students) (88). Being female was identified as a risk factor (30, 89–92) and being male as a protective factor (93) in some German studies. Older age was identified as a risk factor for depressive symptoms by Karing (31) whereas being younger was also found to be a risk factor for heightened distress (94) while another study reported no significant effects on depression, depressive symptoms, or anxiety related to the age of students in German students (32). Also, in line with other recent literature (35), not having received a vaccination and not intending to do so was a significant predictor for lower levels of anxiety, though only marginally so.

Factors with more consistent results across the literature on German students were loneliness and stress as risk factors (29–32) and social support as an overall protective factor (32, 90, 91). Bearing in mind that the considered studies were conducted through different phases of the pandemic while using a heterogenous set of measurements and reporting on various samples of university students, it is crucial to aim for clarification through further research.

### 4.4. Strengths and limitations

This study has several strengths and limitations. First, a major limitation is the cross-sectional design across all three time points. Because of the anonymity of the surveys, it was not possible to identify participants who participated in which (or all of the) surveys. Nevertheless, these nearly identical surveys across three time points over 3 years of pandemic in Germany provide very important insights into mental health outcomes and changes in mental health and related variables in university students. All surveys were conducted in a large German university, including various faculties and students in different stages of their studies and high response rates. However, the generalizability might be limited due to the reliance on a convenience sample, a possible self-selection bias of students with a mental disorder and the fact that only self-report measures had been applied. Furthermore, the sample does not represent the general student population, as certain groups, women, master program students and students without a migratory background were overrepresented. Additionally, due to the observational character of the study, causal inferences cannot be made.

## 5. Conclusion

In general, the results of the present study confirm previous results and results from the international literature that the pandemic had and still has a negative impact on the mental health of university students. The present study broadens this view by the fact that some areas seem to recover quicker, while others seem to increase worryingly. Especially the persistent rise in suicidal ideation from 2020 to 2021 and to 2022, a constant reduction in reported social support and associated perceived loneliness is concerning. The claim for low-threshold and accessible mental health support for university students remains the same as in 2021 (9) and should get more impetus by the present results, regardless of some positive results. Still, online interventions seem to be feasible, evidence-based, easy to implement and promising to fulfill this need of young students. In addition, barriers to accessing the mental health care system should be reduced, not exclusively but especially for young individuals, to prevent the chronification of mental health issues.

## Data availability statement

The raw data supporting the conclusions of this article will be made available by the authors, without undue reservation.

## Ethics statement

The studies involving human participants were reviewed and approved by the Ethics Committee of the Medical Faculty, University Leipzig, Germany. The patients/participants provided their written informed consent to participate in this study.

## Author contributions

LG, EK, SB, and CR-K designed the study. LG performed the statistical analysis. LG and EK drafted the article. LG, EK, SB, TB, JS, and CR-K discussed the results and contributed to the final manuscript. All authors have approved the final manuscript.
